# The Evolving Regulatory Paradigm of AI in MedTech: A Review of Perspectives and Where We Are Today

**DOI:** 10.1007/s43441-024-00628-3

**Published:** 2024-03-25

**Authors:** Karen Zhou, Ginny Gattinger

**Affiliations:** 1 Northeastern University, Toronto, ON Canada; 2 SoftwareCPR, Ottawa, ON Canada

**Keywords:** Artificial intelligence, AI, AI/ML, FDA, WHO, Medical device, Regulatory framework, AI ethics and governance

## Abstract

Artificial intelligence (AI)-enabled technologies in the MedTech sector hold the promise to transform healthcare delivery by improving access, quality, and outcomes. As the regulatory contours of these technologies are being defined, there is a notable lack of literature on the key stakeholders such as the organizations and interest groups that have a significant input in shaping the regulatory framework. This article explores the perspectives and contributions of these stakeholders in shaping the regulatory paradigm of AI-enabled medical technologies. The formation of an AI regulatory framework requires the convergence of ethical, regulatory, technical, societal, and practical considerations. These multiple perspectives contribute to the various dimensions of an evolving regulatory paradigm. From the global governance guidelines set by the World Health Organization (WHO) to national regulations, the article sheds light not just on these multiple perspectives but also on their interconnectedness in shaping the regulatory landscape of AI.

## Introduction

Artificial intelligence (AI) has gained much momentum lately across all sectors of the economy. In the MedTech sector, AI or Machine Learning (ML)-enabled medical devices have the potential to revolutionize healthcare delivery by improving efficiencies and driving better health outcomes. These technologies have also long been under regulatory scrutiny and their regulatory paradigm has been a work in progress. Global regulatory authorities such as the US Food and Drug Administration (FDA) have been leading the way in laying down a regulatory framework for evaluating the safety and effectiveness of AI/ML-enabled medical devices. AI in healthcare or medical AI raises a complex set of regulatory issues that may or may not be mutually exclusive. The issue of transparency in AI/ML-enabled algorithms sets it apart from traditional regulated technologies. Another is the quality of datasets for training AI, which can introduce bias in the output if key demographics are missing. There are also concerns about change management, such as version control and system updates, since AI technology is known for its adaptive nature that allows it to learn, adapt, and change in response to new inputs. These issues are only a few among a list of challenges in the development and deployment of AI/ML-enabled health technologies. Less well articulated in the current literature are the perspectives and contributions of key stakeholders such as the organizations and interest groups that have a significant influence on the regulatory paradigm. It is the objective of the paper to present an overview of the key stakeholders/organizations and their perspectives and contributions in shaping the AI medical device regulatory framework. These groups include industry trade associations, medical associations, patient groups, and global regulatory authorities. Each organization is driven by a unique mission and speaks to a specific domain of expertise. The issues of interest concerning AI/ML-enabled health technologies set the stage for a broader regulatory framework. This paper brings together the perspectives of stakeholders with a significant presence in medical AI regulatory discussions and highlights the current gaps and limitations in this evolving sectoral framework. Notably, much of the regulatory policy discussion takes place through a Western lens such as the case in the USA and EU. It is because as AI innovation hubs, they are further along in the development of a legal and regulatory framework. The focus of this paper is largely through the lens of the AI regulatory stakeholders in the field of healthcare technology in these jurisdictions.

Throughout the paper, the terms AI, AI/ML, and medical AI are used interchangeably to refer to medical technologies enabled with AI functions. The term AI/ML is used by health regulators such as the FDA to incorporate ML, which is a branch of AI that is used to design and train software algorithms to learn from data. In some contexts, such as in healthcare, medical AI is often used interchangeably with AI-enabled health technologies.

## An Ethics and Governance Framework for AI in Health

The World Health Organization (WHO) was the first organization to issue a global report on AI in health, which provides a framework through which regulators, AI developers, and health institutions would engage with AI-based medical technologies [1, 2]. The report represents one of the first calls to action to the stakeholders in AI community to embed six foundational principles for AI regulation and governance: (1) Protecting human autonomy; (2) Promoting human well-being and safety and the public interest; (3) Ensuring transparency, explainability, and intelligibility;(4) Fostering responsibility and accountability; (5) Ensuring inclusiveness and equity; and (6) Promoting AI that is responsive and sustainable [2].

AI, both in general and in the healthcare context, presents numerous ethical challenges that transcend the jurisdiction of any one stakeholder group. Safeguarding the public from potential AI harms is an extension of their traditional role in regulatory policymaking. For health regulatory authorities, which oversee the regulation of medical technologies, the ethical principle of transparency, explainability, and intelligibility is one of the bedrocks of regulatory developments. The “black box” nature of AI technologies poses a significant barrier to assessing the safety and efficacy of these products. Thus, a key factor in regulatory evaluation is the explainability of algorithms to understand how AI arrives at decisions. Another main principle that underpins regulatory decision-making is ensuring inclusiveness and equity. This principle requires that AI for health be designed to be inclusive of age, sex, race, gender, ethnicity, sexual orientation, income, ability, and other protected characteristics. AI technology must be widely shared with all patients regardless of where they are in the world. To this end, regulatory authorities should incentivize AI developers to take measures to minimize bias, which undermines the principle of inclusiveness and equity. Since the WHO report was published in June 2021, the regulation of AI-enabled medical devices has taken priority among the major health regulators and within the International Consortium of Medical Device Regulators (IMDRF), which is discussed in the subsequent section “The Role of the IMDRF.”

### Ethical Challenges in LMICs

To date, AI regulatory development largely takes place in the USA and EU. In the Low- and Middle-Income Countries (LMICs), there are additional ethical considerations that can affect the uptake of AI-enabled medical technologies in healthcare. One consideration is data bias, which is accentuated in these countries. On the technology side, one challenge is the inherent contextual bias due to a lack of quality data to train new algorithms to address profiles of populations in these countries. On the infrastructure side, LMICs also experience a “digital divide” or the uneven distribution of access to information and communication technologies among different groups. For example, women in LMICs are poorly represented compared to men since fewer of them have access to mobile internet and thus have fewer data points.

In LMICs, inadequate regulatory capacity also raises new questions. The reliance on regulatory approval of AI technologies from the USA or EU assumes that criteria used to develop and regulate an AI technology are readily transposable to LMICs. For example, standards, types of data used to train algorithms, and the context in which AI is developed are assumed to apply to LMICs without regard for whether these assumptions are appropriate for those countries. A contextual bias occurs when algorithms may not recommend appropriate or cost-effective treatments for low-resource settings [2].

## Perspectives from the Healthcare Sector

AI-enabled medical technologies also cast the traditional practice of medicine in a new light. Representing healthcare providers, the American Medical Association (AMA) is a proponent of AI-enabled technologies. The incorporation of AI-enabled applications into medical devices has the potential to change health technology and medicine in profound ways [3]. In the view of the AMA, the term “artificial intelligence,” however, is insufficient to capture the role of technologies being developed or in use currently. The reason is that machines have not achieved the capability of understanding and/or learning any single intellectual task at the level of a human being. Instead, the technologies of today are assistive [4]. The term “augmented intelligence” has been introduced to refer to the concept of AI that focuses on its assistive role, which enhances human intelligence rather than replacing it. However, for clinical integration, several key areas remain to be addressed. It is essential to adopt clear consensus standards for the development, management, and use of safe and effective health technology [4].

There are four main questions that must be answered for the clinical integration of AI [5]. They are (1) safety and efficacy; (2) reimbursement; (3) the “black box” nature of AI and allocation of liability; and (4) deployment.

### Ensuring Safety and Efficacy

As in drug development, the premarket standard of safety and efficacy or effectiveness [6] ensures that the AI/ML product not only performs as intended but also generates clinically significant results when used for its intended purpose according to its directions of use. The FDA has had a significant role in developing the premarket regulatory standards for AI/ML product development. FDA regulatory developments in AI/ML will be explored in the later section “National Regulatory Authorities.”

### Reimbursement

The rise in AI/ML-based technologies is evidenced by the increasing number of FDA approvals [7]. The majority of AI/ML devices authorized by the FDA are in radiology followed by cardiovascular and other specialties, such as ophthalmology and neurology. However, in terms of clinical adoption, financing remains the gatekeeper.

AI-enabled technologies, as with any novel technology, raise questions about how much financial risk the healthcare systems and physicians can undertake. According to the AMA, these questions include the cost–benefit of AI-based devices as compared with existing technologies as well as payor coverage [8].

In the USA, the reimbursement process is primarily based on Current Procedural Terminology (CPT) codes created by the AMA which are recognized by both private and public health insurance programs, such as Medicare. To date, only 16 CPT codes have been created for 16 medical AI-based medical procedures [9]. The overall adoption of medical AI-based health technologies is still nascent, with medical devices for procedures associated with coronary artery diseases seeing the most AI usage. One of these AI-based medical devices is HeartFlow FFRCT, used to measure blood flow through the arteries to diagnose and assess coronary artery disease by generating computed tomography (CT)-based 3D modeling of the coronary arteries. Medical procedures utilizing HeartFlow FFRCT are reimbursed by both public and private payors. Diabetic retinopathy medical AI is also growing in usage. An example associated with a CPT code is the IDx-DR, which is used to make a clinical decision without human oversight, including the diagnosis of diabetic retinopathy and macular edema [10, 11].

In the US context, AI adoption varies significantly by geography and socioeconomic factors, as well as the type of hospital (academic hospitals versus general hospitals). Further research is warranted to investigate the barriers to equitable access or wider adoption of AI in healthcare. It is important to note that the rate of AI adoption in the USA is not a reflection of the rest of the world, especially since the USA has both public and private payors as opposed to jurisdictions with a single-payor healthcare system. Nevertheless, due to similarities in technology, the trends in adoption in the USA may also be applicable in other jurisdictions [9].

### “Black Box” Nature and Liability

A third concern to the medical community is the “black box” nature of these tools or the lack of transparency that can be a barrier to presenting a full picture of the risks and benefits of their use. This revolves around algorithmic accountability. Regulatory systems must ensure safe performance and limit liability for those downstream. Liability lies with those best suited to mitigate harm.

In a traditional regulatory framework, validation testing determines whether the product meets user needs and performance specifications. AI raises additional concerns that cannot be addressed by conventional testing. Regulation must also address the “black box” nature of AI, as discussed earlier. The field of medical AI liability remains in flux and involves a complex set of interactions between multiple parties [12]. A regulatory framework that embeds algorithmic transparency is a step toward accountability and can better provide reassurance to healthcare providers and patients. As will be discussed in the section “National Regulatory Authorities,” one of the FDA’s priorities is to support a patient-centered approach that includes the need for a manufacturer’s transparency to users about the functioning of AI/ML-enabled devices to ensure they have a full picture of the benefits, risks, and limitations [13].

### Deployment: Is the Tool Fit for Real-World Use?

Confidence in AI in healthcare depends on the ability to translate proof-of-concept algorithms into clinical care where their predictability and accuracy are put to the test. The term “AI Chasm” has been coined to describe a divide between developing scientifically sound algorithms and their meaningful applications in real settings [14].

A well-documented example of the challenges is the deployment of Google’s AI-based tool for the detection of diabetic retinopathy, Automated Retinal Disease Assessment (ARDA) [15]. In screening more than 200,000 individuals for diabetic retinopathy, Google has provided a first-hand account of the translational challenges that can temper expectations [15].

The team identified 5 main challenges in deployment. First, the quality of the data and labels matters as much as the quantity of data. Second, the availability of multi-disciplinary expertise from a wide range of disciplines is needed to tune the AI model for real-world clinical use. These disciplines include software engineering, data science, user experience, health equity, ethics, and legal, regulatory, and quality, all of which are critical to developing AI models responsibly, keeping human safety and experience at the center of development. Third, how AI models perform in silico does not necessarily reflect their performance in a real-world setting. To that end, retrospective and prospective validation testing are essential for evaluating performance and clinical applicability. Prospective validation testing can reveal unexpected environmental variables. For example, the Google team found that the performance of the algorithm decreased when using low-quality images taken in real-world settings since the AI was trained on high-quality images obtained in controlled settings [15]. The lower-quality images can be attributed to variables, such as inexperienced operators, dirty lenses, and poor lighting. Real-world validation often reveals unexpected issues that were not present during development. Fourth, AI may introduce changes to clinical workflows resulting in adjustments on the part of healthcare providers and patients. [15]. Fifth, proactive post-deployment monitoring is a critical component of the product lifecycle due to anticipated changes, including workflow changes in equipment and patient preparation [15].

## Patient Perspectives

Patients remain a critical input during AI development in healthcare. Amid the ongoing discussion on AI regulation, there is scant literature in the form of systematic studies or policy papers on how patients perceive the benefits of AI. In 2022, the Journal of American Medical Association (JAMA) published a US national survey on how patients view AI applications, including their concerns [16]. Patients mostly had positive views about AI’s ability to improve healthcare; however, the optimism also came with concerns about such issues as data privacy, reduced time with clinicians, and the potential for misdiagnosis. In a 2023 Pew Research Center survey of public views on AI in health and medicine, attitude toward AI can be summed up as cautious optimism [17]. Most US respondents are concerned with the pace of AI technology and its potential for quick adoption by healthcare providers before the risks for patients can be fully understood [17]. In a survey by the European Patients Forum (EPF) to assess AI awareness and perceptions among patient organizations, almost all respondents viewed AI favorably as a technology that has the potential to improve patient outcomes [18]. Respondents’ concerns include biased training data, lack of patient representation in AI development, safety issues attributed to low-quality and unvalidated AI systems, lack of algorithmic transparency, and patients not being informed of AI use in treatment [18].

A regulatory challenge is establishing criteria for the clinical validity of AI technologies. From a patient’s perspective, a regulatory framework establishes not just the safety parameters, but also the guardrails around consent especially when patients are direct participants in AI development. The ethical principle of protecting autonomy requires that the use of AI algorithms in diagnosis and treatment be part of the informed consent process. There is a growing debate over whether or how to inform patients about the use of AI in treatment. How to integrate AI-enabled technologies into the informed consent process is currently a subject of academic inquiry [19]. In one study, it was found that informed consent to use AI/ML devices in the care of patients can be complex given the uncertainties, fears, or possible overconfidence about the performance of AI [20].

## Industry Perspective

As developers and manufacturers of highly innovative technologies, the MedTech industry is a critical stakeholder. The EU has been at the forefront of creating a cohesive regulatory environment for health technology innovations. First, the passage of the EU Medical Device Regulation (MDR) and EU In Vitro Diagnostic Regulation (IVDR) [21, 22] in 2017 overhauled the previous device directives to align the regulatory framework with the pace of innovation. The MDR and IVDR introduced a revamped medical device regulatory framework that called for scrutiny of additional economic operators and incorporated additional clinical evaluation and post-market regulatory considerations. Second, the EU AI Act [23], when it is eventually passed, will provide an additional layer of regulatory expectations for AI technologies beyond the sectoral fundamentals laid down in the MDR/IVDR.

MedTech Europe is the European trade association for the medical technology industry, which includes medical devices, in vitro diagnostics, and digital health. As the voice of the MedTech industry, MedTech Europe is a proponent of AI in medical technologies. It supports AI regulation that provides accessibility to AI in healthcare and does not stifle innovation. For the MDR/IVDR and AI Act to work together, three issues should be addressed [24]:

- Safe, high-quality, and trustworthy AI
- Harmonization and legal certainty
- Innovation

An environment with safe, high-quality, and trustworthy AI in medical technologies provides improved healthcare and patient outcomes. The embrace of AI technologies depends on the safety, transparency, and explainability of these technologies. From an industry perspective, harmonization of rules avoids the fragmentation between horizontal and sectoral regulations represented by the AI Act and EU MDR/IVDR, respectively. Moreover, due to the fast-paced nature of AI development in medical AI, a legal and regulatory framework that supports innovation offers the fundamental structure for the development of AI-enabled technologies.

Unlike in the EU, the regulation of medical AI in North America (USA and Canada) has largely been within the ambit of sectoral regulators such as the FDA and Health Canada, while a general AI law in both countries is still being debated or gradually wending its way through the political process [25, 26]. Without an overarching AI legal framework, the regulatory landscape for the industry has been a patchwork of sectoral regulatory policy and corporate commitments to responsible AI. Organizations at the individual level have developed internal ethical principles to guide the development of AI technologies [27, 28]. Common principles are ensuring patient data privacy and security, algorithmic transparency, and bias mitigation.

## The Role of the IMDRF

The IMDRF consists of a voluntary group of medical device regulators from around the world who work together on medical device regulatory harmonization and convergence initiatives. Under the aegis of an international forum, many foundational regulatory principles have been established and incorporated into national regulatory frameworks. The current members of IMDRF include Canada, USA, Australia, Japan, and other jurisdictions with a few organizations sitting as observers, such as the WHO. It is through IMDRF’s Working Groups that premarket and post-market regulatory principles are defined and established in consensus documents, which, while non-binding, lay down principles for adoption by the national regulatory authorities. One of the current Working Groups is focused on AI/ML. Having recognized the opportunities of AI/ML in advancing healthcare and the regulatory considerations, the purpose of the aptly named Artificial Intelligence/Machine Learning-enabled Working Group is to reach a regulatory consensus in the AI/ML sector. The goal is to form a consensus on the topic of Good Machine Learning Practice (GMLP) to provide internationally harmonized principles to support the development of safe and effective AI/ML-enabled medical devices. In an inaugural publication on AI, IMDRF established the foundational terms and definitions to be applied across the Total Product Life Cycle (TPLC) to promote consistency and support global harmonization [29]. To date, Health Canada is the only known major regulatory authority to have adopted IMDRF terms and definitions applicable to AI/ML-enabled medical devices [30].

## National Regulatory Authorities

National health regulators are at the center of the evolving regulatory paradigm. Regulators must balance the need to facilitate innovation with their responsibility to safeguard public health. The regulatory challenge posed by AI-based technologies throws this fine balance into sharp relief. At the national level, the rapid advancement of AI-enabled medical technologies has been the impetus behind recent regulatory developments. The FDA has been leading the development of an AI regulatory framework in medical devices and in effect and has been gradually paving the way toward a sectoral model that will serve as a reference point globally. This effort can be traced back to 2019 when the agency released a discussion paper proposing a framework for modifications to AI/ML-based medical devices, which recognizes the iterative nature of AI/ML devices while assuring safety and effectiveness [31]. Following the 2019 discussion paper and stakeholder feedback, the FDA developed an AI/ML Software as Medical Device Action Plan in January 2021 (“Action Plan”) in keeping with their commitment to support innovation in the regulation of medical device software and digital health technologies [13].

One priority in the Action Plan is to strengthen the FDA’s commitment to encouraging the harmonization of a set of AI/ML best practices, such as GMLP. Indeed, in October 2021, the FDA, Health Canada, and the United Kingdom (UK)’s Medicines and Healthcare products Regulatory Agency (MHRA) published 10 guiding principles to inform the development of GMLP [32]. Similar to a quality management system standard, GMLP represents an industry best practice to address the unique nature of AI/ML development.

Another priority is the development of a Predetermined Change Control Plan (PCCP) to guide AI developers on documentation, validation, and impact assessment of algorithmic changes to their AI/ML products and a draft version was published in April 2023 [33]. In October 2023, the FDA, Health Canada, and the MHRA jointly put forth 5 guiding principles for predetermined change control plans [34].

The FDA’s Action Plan is also significant for capturing the FDA’s commitment on two fronts: (1) supporting a patient-centered approach to incorporate transparency to use and (2) supporting the development of regulatory science methods to evaluate and address algorithmic bias to promote algorithm robustness [35]. In order to promote a patient-centered approach to the development of AI/ML-based technologies, the agency has engaged with various stakeholders on where to clarify its position on the transparency of AI/ML-enabled medical devices. For example, in an October 2021 public workshop, the FDA sought feedback to identify unique regulatory considerations for achieving transparency and collected input on what information would be helpful to be included in a manufacturer’s labeling of AI/ML-enabled medical devices [35].

Acknowledging issues of bias and generalizability in the context of opaque algorithms, the FDA also commits to supporting regulatory science initiatives that seek to identify and address bias in algorithms. Regulatory science will likely have an increasing role in the development of tools and approaches to the evaluation of these new technologies.

At the global level, the development of a regulatory framework to adapt to AI-enabled medical technologies is also underway. Health Canada, at the time of writing, is working on a premarket AI regulatory framework [30]. In Asian countries, both Japan and China have worked toward a regulatory foundation tailored to AI-enabled medical technologies. Japan has engaged in the process of adopting a regulatory framework to approve AI/ML-based medical devices where performance changes over time, similar to the FDA’s PCCP regulatory requirements [36]. China has worked on various initiatives to develop a regulatory framework and published several AI-related proposals and an AI-based medical device standards system [37, 38].

For a summary of FDA’s and Health Canada’s published AI/ML medical device regulatory policy and guidance by the end of 2023, refer to Table 1.

**Table 1:** Summary AI/ML Medical Device Regulatory Policy and Guidance 2019–2023

Regulatory Development	Month/Year
**USA**	
Proposed Regulatory Framework for Modifications to Artificial Intelligence/Machine Learning (AI/ML)-Based Software as a Medical Device (SaMD)—Discussion Paper and Request for Feedback	April 2019
Artificial Intelligence/Machine Learning (AI/ML)-Based Software as a Medical Device (SaMD) Action Plan	January 2021
Good Machine Learning Practice for Medical Device Development: Guiding Principles (FDA, Health Canada, MHRA)	October 2021
Marketing Submission Recommendations for a Predetermined Change Control Plan for Artificial Intelligence/Machine Learning (AI/ML)-Enabled Device Software Functions—Draft Guidance for Industry and Food and Drug Administration Staff	April 2023
Predetermined Change Control Plans for Machine Learning-Enabled Medical Devices: Guiding Principles (FDA, Health Canada, MHRA)	October 2023
**Canada**	
Draft Guidance: Pre-market guidance for machine learning-enabled medical devices	September 2023

## Standards Development Organizations

Standards Development Organizations (SDOs) are another pillar in AI development. Medical device manufacturers generally adhere to published standards when developing their products as they are considered the gold standard to follow. In any industry, standards are developed based on existing best practices and internally agreed upon by groups of experts from an array of experiences and backgrounds. The International Organization for Standardization (ISO), along with the International Electrotechnical Commission (IEC) and the Institute of Electrical and Electronics Engineers (IEEE), are among the well-recognized organizations that publish international standards in many disciplines. In the medical device industry, standards can be officially “recognized” by regulatory bodies, such as the FDA and Health Canada, particularly if a representative of a particular regulatory body is part of the committee developing the standard. Adhering to a standard recognized by a regulatory body may help streamline the review process for medical device approval.

The IEC and ISO have joined forces to form a joint technical committee, known as ISO/IEC JTC 1/SC 42 (“SC 42”), to develop international standards for AI/ML [39]. SC 42 is described as the first international standards committee to utilize a holistic approach that considers the entire AI/ML ecosystem, considering elements, such as business, regulatory, and policy requirements, application domain needs, as well as ethical and societal concerns. At the time of this writing, SC 42 has already released over twenty standards, including topics of data quality, AI system life cycle processes, and guidance for human oversight of AI systems, and has over thirty standards currently under development [40]. While these standards are industry agnostic, they can be used as a starting point for standardization for AI/ML-based medical devices.

Another major institution involved in developing standards is the Association for the Advancement of Medical Instrumentation (AAMI), an American-based nonprofit organization with over five decades of history and comprises over 10,000 professionals representing clinicians, the healthcare industry, and regulatory agencies. AAMI provides practical guidance and support to the healthcare technology industry in the form of training and consensus standard publications, both national and international. While AAMI is more US-based as compared to ISO, IEC, and IEEE’s international nature, AAMI’s most recent contribution to the AI/ML guidance development is its 2022 consensus report on performing risk management for medical devices incorporating AI/ML entitled *Guidance on the Application of ISO 14971 to Artificial Intelligence and Machine Learning* (AAMI CR34971:2022) [41]. The consensus report (CR) was subsequently upgraded to a Technical Information Report (TIR) [42], which is more technical than a consensus report and provides in-depth methodologies to guide technical development. The purpose of this report is to guide practical applications of the well-established and widely used risk management standard ISO 14971:2019 [43] as it pertains to AI/ML-based devices. To date, AAMI CR34971:2022 (now TIR 34971:2023) is the first AI-focused document to be incorporated by the FDA as a Recognized Consensus Standard [44, 45].

## Common Themes and Future Directions

Regulation of AI-enabled health technologies requires a convergence of stakeholder views from different perspectives. Regulators respond to concerns emanating from the general society, including patient groups, industry, and healthcare providers. Several common themes emerge from the stakeholders. They can be summarized as a need for trustworthy AI and regulatory certainty.

### Trustworthy AI

One common thread in the stakeholders’ perspectives is building trustworthy AI that merits the confidence of healthcare providers and patients. Trustworthy AI should be lawful, ethical, and robust [46]. A wide adoption of AI-enabled technologies must be built on trust. This trust comes from building compliant and robust technologies in which ethics is integrated into every stage of the product life cycle.

To ensure ethics is built into AI-enabled medical technologies, regulatory science will continue to drive the development and application of scientific and technical approaches to evaluate AI-based products. As discussed above, regulatory science will contribute to the FDA’s development of an AI regulatory paradigm. One active area of research is bias mitigation in AI-enabled medical technologies. An example is a bioethical analysis of the AI life cycle premised on the FDA’s TPLC framework [31, 47, 48]. The pipeline model framework provides a systematic appraisal of the AI model from early development through deployment by working through the sources and impacts of bias on health equity, which will inform stakeholders, including regulators, healthcare providers, as well as payors [47, 48].

Trustworthy AI is also robust, which means that any AI-enabled technology also meets rigorous technical standards. To that end, SDOs will have an increasingly prominent role in fostering and facilitating the innovative development of AI-based medical devices. They contribute to regulatory development as we saw with the adoption of AAMI CR34971:2022 by the FDA. Although it may seem that the efforts and focus of each working group are segregated, the hope is that these groups will benefit from the agility of their small sizes as well as their “grassroots” and nimble nature to produce much-needed structure and solutions for manufacturers in the innovative space, and in turn, these isolated efforts combined will form a cohesive holistic and practical framework for the industry. Like the diversity in medical devices in general, the complexity and nature of AI-based medical devices will vary from one to another and therefore, any best practices or standardization set forth by SDOs are only guidelines. Ultimately, it is the responsibility of each manufacturer to adopt the methods and practices to ensure the AI-based devices are not only compliant with laws and regulations but also robust.

### Regulatory Certainty

Global health authorities have made significant strides in the harmonization of regulatory principles. There is consensus on what a medical device framework in the era of digital health looks like. There is a stronger impetus in the age of AI to align on a common set of global principles going forward. Under the leadership of the FDA, a sectoral medical device framework tailored to AI-enabled technologies is coming into focus. As this paper has emphasized, regulatory development in the AI space largely occurs in North America and the EU. In the long term, perspectives from the LMICs as well as the advanced economies of Asia (an analysis is beyond the scope of this paper) would need to be integrated into a coherent global framework from a health equity perspective. That the health authorities of the USA, EU, and the UK have developed a set of harmonized principles for AI development is an encouraging first step from a global harmonization perspective. However, these technical considerations will eventually need to be complemented by global guidelines on embedding ethics considerations during AI development.

## Conclusion

Technological advancements tend to outpace the development of corresponding legal and regulatory frameworks due to technology's agnostic nature. AI is an exemplar of these advancements. The stakeholder groups surveyed in this paper all contribute to the AI regulation debate. An important question is whether the groups writing the rules of AI governance now, largely through a Euro-American lens, are paving the way toward an inclusive and equitable framework. Ultimately, it is the choices that policymakers and regulatory authorities make that determine the direction of AI and its impact on humanity.

## Data Availability

No data used in this article.

## References

[1] World Health Organization. WHO Issues First Global Report on Artificial Intelligence (AI) in Health. 2021. https://www.who.int/news/item/28-06-2021-who-issues-first-global-report-on-ai-in-health-and-six-guiding-principles-for-its-design-and-use

[2] World Health Organization. Ethics and Governance of Artificial Intelligence for Health: WHO Guidance. 2021. https://www.who.int/publications/i/item/9789240029200

[3] Lubell J. As Health Care AI Advances Rapidly, What Role for Regulators? American Medical Association. 2023 https://www.ama-assn.org/practice-management/digital/health-care-ai-advances-rapidly-what-role-regulators

[4] Association for the Advancement of Medical Instrumentation Artificial Intelligence versus Augmented Intelligence: What’s in a Word? September 21, 2022. https://array.aami.org/content/news/artificial-intelligence-versus-augmented-intelligence-s-word

[5] American Medical Association. Augmented Intelligence in Health Care. 2018. https://www.ama-assn.org/system/files/2019-08/ai-2018-board-report.pdf

[6] Determination of Safety and Effectiveness 21 C.F.R. Sect. 860.7(2018).

[7] US FDA. Artificial Intelligence and Machine Learning (AI/ML)-Enabled Medical Devices. 2023. https://www.fda.gov/medical-devices/software-medical-device-samd/artificial-intelligence-and-machine-learning-aiml-enabled-medical-devices

[8] Association for the Advancement of Medical Instrumentation (AAMI). Artificial Intelligence: The Trust Issue. 2023. https://array.aami.org/pb-assets/pdf/MDSiF-AI_ExecSumm%20040323-1680544083.pdf

[9] Wu K, Wu, E, Theodorou, B, et al. Characterizing the Clinical Adoption of Medical AI Devices through U.S. Insurance Claims. NEJM AI. November 9, 2023. https://onepub-media.nejmgroup-production.org/ai/media/b35da8b4-b078-492b-ae20-bf938063e91f.pdf

[10] Digital Diagnostics. IDx-DR. 2023 https://www.digitaldiagnostics.com/products/eye-disease/idx-dr-eu/

[11] US FDA. FDA Permits Marketing of Artificial Intelligence-Based Device to Detect Certain Diabetes-Related Eye Problems. April 11, 2018. https://www.fda.gov/news-events/press-announcements/fda-permits-marketing-artificial-intelligence-based-device-detect-certain-diabetes-related-eye

[12] Price, WN, Gerke, S, Cohen, IG. Liability for Use of Artificial Intelligence in Medicine. 2022. Law & Economics Working Papers. 241. https://repository.law.umich.edu/law_econ_current/241

[13] US FDA. Artificial Intelligence/Machine Learning (AI/ML)-Based Software as a Medical Device (SaMD) Action Plan. 2021. https://www.fda.gov/media/145022/download

[14] Keane PA, Topol EJ (2018). With an eye to AI and autonomous diagnosis. Npj Digit Med.

[15] Widner K, Virmani S, Krause J (2023). Lessons learned from translating AI from development to deployment in healthcare. Nat Med.

[16] Khullar D, Casalino LP, Qian Y (2022). Perspectives of patients about artificial intelligence in health care. JAMA Netw Open.

[17] Tyson A, Pasquini G, Spencer A, et al. 60% of Americans Would Be Uncomfortable with Provider Relying on AI in Their Own Health Care. Pew Research Center. 2023 https://www.pewresearch.org/science/2023/02/22/60-of-americans-would-be-uncomfortable-with-provider-relying-on-ai-in-their-own-health-care/

[18] Survey Results: Assessing AI Awareness and Perceptions Among Patient Organisations [Internet]. European Patients Forum. 2023. Available from: https://www.eu-patient.eu/ai-knowledgehub/epf_aiwork/survey-assessing-ai-awareness-and-perceptions-among-patient-organisations/

[19] Cohen IG (2020). Informed consent and medical artificial intelligence: what to tell the patient?. SSRN Electron J.

[20] Schiff D, Borenstein J (2019). How should clinicians communicate with patients about the roles of artificially intelligent team members?. AMA J Ethics.

[21] European Union. Regulation (EU) 2017/745. https://eur-lex.europa.eu/eli/reg/2017/745/oj

[22] European Union. Regulation (EU) 2017/746. https://eur-lex.europa.eu/eli/reg/2017/746/oj

[23] European Union. Proposal for a Regulation of The European Parliament and of the Council Laying Down Harmonised Rules on Artificial Intelligence (Artificial Intelligence Act) And Amending Certain Union Legislative Acts. https://eur-lex.europa.eu/legal-content/EN/TXT/?uri=celex%3A52021PC0206

[24] Key messages on the proposal for a Regulation laying down harmonised rules on artificial intelligence MedTech Europe views on the Artificial Intelligence Act. 2022. https://www.medtecheurope.org/wp-content/uploads/2022/12/221202-key-messages-on-the-ai-act_final.pdf

[25] US Government. Blueprint for an AI Bill of Rights. 2023 https://www.whitehouse.gov/ostp/ai-bill-of-rights/

[26] Government of Canada. C-27: An Act to enact the Consumer Privacy Protection Act, the Personal Information and Data Protection Tribunal Act and the Artificial Intelligence and Data Act and to make consequential and related amendments to other Acts. https://www.parl.ca/legisinfo/en/bill/44-1/c-27

[27] The Medtronic AI Compass. https://www.medtronic.com/us-en/our-company/key-facts/artificial-intelligence-compass.html

[28] Philips AI principles. https://www.philips.com/a-w/about/artificial-intelligence/philips-ai-principles.html

[29] International Medical Device Regulators Forum. Machine Learning-enabled Medical Devices: Key Terms and Definitions.2022. https://www.imdrf.org/sites/default/files/2022-05/IMDRF%20AIMD%20WG%20Final%20Document%20N67.pdf

[30] Health Canada. Draft guidance: Pre-market guidance for machine learning-enabled medical devices. 2023. https://www.canada.ca/en/health-canada/services/drugs-health-products/medical-devices/application-information/guidance-documents/pre-market-guidance-machine-learning-enabled-medical-devices.html

[31] US FDA. Proposed regulatory framework for modifications to artificial intelligence/machine learning (AI/ML)-based software as a medical device (SaMD) —Discussion paper and request for feedback. 2019 https://www.fda.gov/media/122535/download

[32] US FDA. Good Machine Learning Practice for Medical Device Development: Guiding Principles. 2021. https://www.fda.gov/media/153486/download

[33] US FDA. Marketing Submission Recommendations for a Predetermined Change Control Plan for Artificial Intelligence/Machine Learning (AI/ML)-Enabled Device Software Functions - Draft Guidance for Industry and Food and Drug Administration Staff. 2023. https://www.regulations.gov/document/FDA-2022-D-2628-0002

[34] US FDA. Predetermined Change Control Plans for Machine Learning-Enabled Medical Devices. 2023. https://www.fda.gov/media/173206/download?attachment

[35] US FDA. Virtual Public Workshop - Transparency of Artificial Intelligence/Machine Learning-enabled Medical Devices. 2021. https://www.fda.gov/medical-devices/workshops-conferences-medical-devices/virtual-public-workshop-transparency-artificial-intelligencemachine-learning-enabled-medical-devices 10.1038/s41746-023-00992-8 PMC10810855 38273098

[36] The Tokyo Foundation for Policy Research. Delivering on the Promise of Medical AI: Can Japan’s Regulators Keep Pace? (Part 1). May 18, 2022. https://www.tokyofoundation.org/research/detail.php?id=892

[37] King H. China’s digital health regulatory framework for SaMD. Regulatory Focus. November 30, 2022. https://www.raps.org/news-and-articles/news-articles/2022/11/chinas-digital-health-regulatory-framework-for-sam

[38] Asia-Pacific Medical Technology Association. Digital Health Regulation in Asia-Pacific (China and Korea). October 2021. https://apacmed.org/content/uploads/2021/10/DHC-Regulatory-Paper_China-Korea.pdf

[39] International Electrotechnical Commission. SC 42 Strategic Business Plan Artificial Intelligence. https://assets.iec.ch/further_informations/21538/ISO-IECJTC1_N15992_SC%2042%20Business%20Plan%202022.pdf?0914T03

[40] International Standards Organization. ISO/IEC JTC 1/SC 42 Artificial intelligence. https://www.iso.org/committee/6794475.html

[41] Association for the Advancement of Medical Instrumentation. New AAMI Consensus Report: Guidance on Risk Management for AI, ML. April 28, 2022. https://array.aami.org/content/news/new-aami-consensus-report-guidance-risk-management-ai-ml

[42] Association for the Advancement of Medical Instrumentation (AAMI). AAMI TIR34971:2023; Application of ISO 14971 to Machine Learning in Artificial Intelligence—Guide. 10.2345/9781570208669

[43] International Standards Organization. ISO 14971:2019 Medical devices—Application of Risk Management to Medical Devices. https://www.iso.org/standard/72704.html

[44] Association for the Advancement of Medical Instrumentation. FDA Recognizes First AI-Focused Document, AAMI CR34971:2022, in List of Consensus Standards. January 31, 2023. https://array.aami.org/content/news/u-s-fda-recognizes-first-artificial-intelligence-guidance-among-updated-list

[45] US FDA. Recognized Consensus Standards: Medical Devices. 2023. https://www.accessdata.fda.gov/scripts/cdrh/cfdocs/cfStandards/detail.cfm?standard__identification_no=43891

[46] European Commission. Ethics guidelines for trustworthy AI. April 8, 2019. https://digital-strategy.ec.europa.eu/en/library/ethics-guidelines-trustworthy-ai

[47] Abràmoff MD, Tarver ME, Loyo-Berrios NL (2023). Considerations for addressing bias in artificial intelligence for health equity. npj Digit. Med..

[48] Char DS, Abràmoff MD, Feudtner C (2020). Identifying ethical considerations for machine learning healthcare applications. Am J Bioeth.

